# Supplementary material for: The Systems Biology Research Tool: evolvable open-source software
Source: BMC Syst Biol. 2008 Jun 29;2:55. doi: 10.1186/1752-0509-2-55 (PMC2446383; doi:10.1186/1752-0509-2-55)
Supplement: Additional file 1 — SBRT Archive. An archive of the current version of the Systems Biology Research Tool. [file 1752-0509-2-55-S1.zip › sbrt-1.4.0/doc/developers_guide/api/sbrt/kernel/fba/comp/package-use.html]

Uses of Package sbrt.kernel.fba.comp


|  |  |  |
| --- | --- | --- |
| **Overview** \| **Package** \| Class \| **Use** \| **Tree** \| **Deprecated** \| **Index** \| **Help** | |  |
| PREV   NEXT | **FRAMES**    **NO FRAMES**     **All Classes** |  |


---


## **Uses of Package sbrt.kernel.fba.comp**

| Packages that use sbrt.kernel.fba.comp | |
| --- | --- |
| **sbrt.kernel.fba.comp** | Provides classes and interfaces for performing Flux Balance Analysis computations. |
| **sbrt.kernel.fba.proc** | Provides classes and interfaces for executing Flux Balance Analysis processes. |
| **sbrt.shell.io.fba** | Provides classes and interfaces for reading and writing files relevant to Flux Balance Analysis. |
| **sbrt.shell.mng.fba** | Provides classes and interfaces for creating Flux Balance Analysis process managers. |

Classes in sbrt.kernel.fba.comp used by sbrt.kernel.fba.comp

****AbstractCvComputer****
  
          This abstract class is a skeleton implementation of `FluxCvComputer`.

****AbstractFluxIntervalComputer****
  
          This abstract class is a skeleton implemenation of
`FluxIntervalComputer`.

****CdHarFluxVectorComputer.Results****
  
          This class is used to represent the results of a coordinate-direction
hit-and-run flux vector computation.

****CycleComputer****
  
          This interface is used to represent algorithms for identifying steady state
cycles (Type III extreme pathways) in stoichiometric networks.

****ExtremeCurrentComputer****
  
          This interface is used to represent extreme current computers.

****FbaOptComputer****
  
          This class is the workhorse of all Flux Balance Analysis optimizations.

****FbaOptComputer.Results****
  
          This class is used to store the results of an FBA optimization
problem.

****FluxCvComputer****
  
          This interface is used to represent a flux constraint variation computer.

****FluxIntervalComputer****
  
          This interface is used to represent flux interval computers.

****FluxomeReducer****
  
          This interface is used to represent algorithms that reduce the size of fluxomes.

****InitialPointComputer.Results****
  
          This class is used to represent the results of a

Classes in sbrt.kernel.fba.comp used by sbrt.kernel.fba.proc

****CdHarFluxVectorComputer****
  
          This class is used to compute randomly distributed vectors within flux space.

****CdHarFluxVectorComputer.Results****
  
          This class is used to represent the results of a coordinate-direction
hit-and-run flux vector computation.

****CycleComputer****
  
          This interface is used to represent algorithms for identifying steady state
cycles (Type III extreme pathways) in stoichiometric networks.

****ExtremeCurrentComputer****
  
          This interface is used to represent extreme current computers.

****FbaOptComputer****
  
          This class is the workhorse of all Flux Balance Analysis optimizations.

****FbaOptComputer.Results****
  
          This class is used to store the results of an FBA optimization
problem.

****FluxCapComputer****
  
          This class is used to compute *flux caps* for use in Mahadevan-Schilling
flux interval computations.

****FluxCvComputer****
  
          This interface is used to represent a flux constraint variation computer.

****FluxIntervalComputer****
  
          This interface is used to represent flux interval computers.

****FluxomeReducer****
  
          This interface is used to represent algorithms that reduce the size of fluxomes.

****InitialPointComputer****
  
          This class is used to compute initial points for coordinate-direction
hit-and-run random flux vector algorithms.

****InitialPointComputer.Results****
  
          This class is used to represent the results of a

****RxnDeletionComputer****
  
          This class is used to compute the effect of deleting reactions in a
fluxome.

Classes in sbrt.kernel.fba.comp used by sbrt.shell.io.fba

****FbaOptComputer.Results****
  
          This class is used to store the results of an FBA optimization
problem.

Classes in sbrt.kernel.fba.comp used by sbrt.shell.mng.fba

****CdHarFluxVectorComputer****
  
          This class is used to compute randomly distributed vectors within flux space.

****ExtremeCurrentComputer****
  
          This interface is used to represent extreme current computers.

****FbaOptComputer****
  
          This class is the workhorse of all Flux Balance Analysis optimizations.

****FbaOptComputer.Results****
  
          This class is used to store the results of an FBA optimization
problem.

****FbaOptException****
  
          This class is used to indicate that an optimal solution could not be found
or does not exist.

****FluxRangeException****
  
          This class is used to indicate that the range of flux interval has exceeded
a defined cutoff value.

****InvalidFluxVectorException****
  
          This class is used to indicate that a flux vector is invalid.

****MsFluxIntervalComputer****
  
          This class is used to compute Mahadevan-Schilling flux intervals.

****ReverseRxnFluxIntervalComputer****
  
          This class is used to compute flux intervals while constraining the fluxes of
reverse reactions to their minimum.

****RxnDeletionComputer****
  
          This class is used to compute the effect of deleting reactions in a
fluxome.

****SimpleFluxIntervalComputer****
  
          This class is used to compute flux intervals in the simplest possible way.

---


|  |  |  |
| --- | --- | --- |
| **Overview** \| **Package** \| Class \| **Use** \| **Tree** \| **Deprecated** \| **Index** \| **Help** | |  |
| PREV   NEXT | **FRAMES**    **NO FRAMES**     **All Classes** |  |


---
